# Clinical and Molecular Characteristics of Megakaryocytes in Myelodysplastic Syndrome

**DOI:** 10.1055/s-0044-1787752

**Published:** 2024-06-10

**Authors:** Fangxiu Luo, Jialu Zhao, Yubao Chen, Zhenping Peng, Ran An, Yeling Lu, Jiaming Li

**Affiliations:** 1 Department of Laboratory Medicine, Ruijin Hospital, Shanghai Jiao Tong University School of Medicine, Shanghai, China; 2 Transfusion Department, Ruijin Hospital, Shanghai Jiao Tong University School of Medicine, Shanghai, China; 3 Department of Pathology, Ruijin Hospital, Shanghai Jiao Tong University School of Medicine, Shanghai, China; 4 Department of Hematology, Ruijin Hospital, Shanghai Jiao Tong University School of Medicine, Shanghai, China

**Keywords:** MDS, CD34, MK, CD62P, emperipolesis, *TUBB1*

## Abstract

**Objective** Myelodysplastic syndrome (MDS) is a malignant clonal disorder of hematopoietic stem cells which is characterized by morphologic dysplasia. However, the pathological characteristics of megakaryocytes (MKs) in MDS patients with gene mutation are not well established.

**Methods** Bone marrow MK specimens from 104 patients with primary MDS were evaluated, and all patients were distributed into two groups according to gene mutation associated with functional MKs. The morphologic and cellular characteristics of MKs and platelets were recorded and compared.

**Results** The more frequently mutated genes in MDS patients were *TUBB1* (11.54%), *VWF* (8.65%), *NBEAL2* (5.77%), and the most common point mutation was *TUBB1* p.(R307H) and p.(Q43P). Patients with MK mutation showed a decrease in adenosine diphosphate-induced platelet aggregation, high proportion of CD34 ^+^ CD61 ^+^ MKs (10.00 vs. 4.00%, *p* = 0.012), and short overall survival (33.15 vs. 40.50 months, *p* = 0.013). Further, patients with a higher percent of CD34 ^+^ CD61 ^+^ MKs (≧20.00%) had lower platelet counts (36.00 × 10 ^9^ /L vs. 88.50 × 10 ^9^ /L, *p* = 0.015) and more profound emperipolesis ( *p* = 0.001). By analyzing RNA-sequencing of MKs, differentially expressed mRNA was involved in physiological processes including platelet function and platelet activation, especially for MDS patients with high percent of CD34 ^+^ CD61 ^+^ MKs. The high levels of expression of CD62P, CXCL10, and S100A9 mRNA, shown by RNA sequencing, were validated by PCR assay.

**Conclusion** High proportion of CD34 ^+^ CD61 ^+^ MKs was a poor prognostic factor in MDS patients with MK mutation. CD62P, CXCL10, and S100A9 may be the potential targets to evaluate the molecular link between gene defects and platelet function.

## Introduction

Myelodysplastic syndrome (MDS) is a malignant clonal disorder of hematopoietic stem cells, characterized by ineffective hematopoiesis, single or multilineage dysplasia, and risk of progression to acute leukemia. Both morphological and functional defects of platelets have been observed in MDS patients, due to the dysplasia of megakaryocytes (MKs). 1 2 Megakaryocytic proliferation/differentiation is a complex process that involves up/down expression of signaling molecules in the bone marrow (BM) microenvironment. Acquired or inherited mutations affecting the of MK development have been identified. *RUNX1* mutation is associated with thrombocytopenia, leading to the increased CD34 expression on MKs. *TUBB1* or GATA1 mutation contribute to a selective advantage for the CD34 ^+^ clones and abnormal platelet physiology. 3 4 5 6 In general, CD34 expression on the megakaryocytic lineage is limited to promegakaryoblast or megakaryoblast and declines progressively throughout cell maturation. 7 Recent evidence suggests that a high level of CD34/CD61 dual positive MKs (CD34 ^+^ CD61 ^+^ MKs) is associated with lower platelet count, cytogenetic abnormalities, and shorter survival. 8 9

Clinically, low platelet count is often related to bleeding complications in MDS patients. However, because classic “platelet generating” MKs are altered by gene mutation, it is conceivable that platelet function abnormalities may play a role as well. A few studies have reported that, mainly with platelet activation and aggregation, many MDS patients demonstrated impaired platelet phenotypes and reduced functions. 10 11 Although platelet transfusions have greatly reduced the incidence of major hemorrhagic complications, refractoriness to infused platelets becomes a major clinical problem. In this study, we will investigate the pathological characteristics of BM MKs and their relationship with driver gene mutation, aiming to find the potential molecular targets in MDS.

## Methods

### Patients

Patients with MDS were identified at Ruijin Hospital, Shanghai Jiao Tong University from May 2019 to December 2023. All study participants were diagnosed and classified according to the World Health Organization (WHO) 2022 classification. 12 The prognostic impact was evaluated with the International Prognostic Scoring Systems-Revised (IPSS-R). 13 Peripheral blood (PB) collection and BM biopsies were performed on cases after obtaining written consent. Patient-matched germline reference samples such as oral mucosal cells and hair with hair follicles were also harvested. Clinical data related to age, blood count, and BM biopsy at diagnosis were collected from patients' medical records. The overall survival (OS) was evaluated as disease outcomes, and events were defined as death. All survival end points were censored at the date of last follow-up when progression or death was not observed. The research protocol was approved by the Ethic Committees of Ruijin Hospital, Shanghai Jiao Tong University School of Medicine.

### Morphological Evaluation and Immunocytochemistry

All patients had representative BM biopsies, PB and BM aspirate smears available for evaluation. BM smears were stained with Wright's-Giemsa (Baso Diagnostics Inc, Zhuhai, China) and observed by light microscopy (BX41, Olympus Corporation, Tokyo, Japan). For morphologic dysplasia, features of dysmegakaryopoiesis had to be present in at least 10% of the cells of the respective lineage. Multilineage dysplasia involved at least 10% of the cells in two or more lineages. Immunohistochemical staining for CD34 (1:160 dilution; Dako, Glostrup, Denmark) and CD61 (1:100 dilution; Dako, Copenhagen, Denmark) in MKs was performed on formalin-fixed paraffin-embedded BM biopsy sections after heat-induced antigen retrieval using the avidin-biotin peroxidase technique. One hundred MKs or all MKs in the biopsy (if MKs <100) were counted. Percentage of CD34 ^+^ CD61 ^+^ MKs was calculated as CD34 ^+^ CD61 ^+^ MKs/total MKs, CD34 ^+^ CD61 ^+^ MKs ≥20% were considered as high-level or positive, and cases with CD34 ^+^ CD61 ^+^ MKs <20% were considered as negative.

Enzyme-Linked Immunosorbent AssayBM samples were harvested from MDS patients. BM fluids were obtained by centrifugation of 3,500 × g for 15 minutes. Levels of S100A9 proteins in the BM fluids were measured using human S100A9 enzyme-linked immunosorbent assaykits (R&D Systems) according to the manufacturer's instructions.

### Platelet Phenotyping and Function Analysis

Washed platelets in Tyrode's buffer at a concentration of 3 × 10 ^8^ /mL was performed as previously described. 14 Platelet aggregation was analyzed at 37°C using a Platelet Aggregation Profiler (Chrono-Log, Havertown, Pennsylvania, United States). Three independent experiments were performed to ensure the accuracy of the experimental results obtained. Washed platelets were preincubated with peptides (250 μM) at 37°C for 30 minutes, stimulated for 3 minutes with or without thrombin (0.1 U/mL) at 37°C, and immediately fixed with 1% paraformaldehyde. The fixed platelets were labeled with a PE-CD62P and FITC-Annexin V antibody (BD, Franklin Lakes, New Jersey, United States) at concentrations recommended by the manufacturers. The same conjugated nonspecific isotype IgG was used as a negative control. CD62P and Annexin V surface expression were analyzed by flow cytometry. All the samples were analyzed or stored properly within 2 hours of sampling as recommended to avoid significant artifacts in platelet analysis and the release of cell microparticles due to storage.

### DNA Extraction and Targeted Next-Generation Sequencing of Megakaryocytes

BM mononuclear cells were obtained by centrifugation on a Ficoll–Hypaque at a density gradient of 1,500 × g for 25 minutes and then washed three times in phosphate-buffered saline. Genomic DNA was isolated from bone marrow mononuclear cells and was extracted by Qiagen blood extraction kit (Qiagen, Hilden, Germany) following the manufacturer's protocol. DNA quality was assessed by agarose gel electrophoresis and NanoDrop 2000 spectrophotometer (Thermo Fisher Scientific, Wilmington, Delaware, United States). Targeted amplicon-based NGS of up to 20 MK genes were performed. DNA samples were subjected to targeted genome sequencing using Illumina HiSeq2000. As previously described, 15 we established filters for the pathogenic versus nonpathogenic call algorithm to determine clinically actionable pathogenic alterations and to exclude benign variants or polymorphisms.

### RNA Isolations and RNA-Sequencing of Megakaryocytes

Ten milliliters of fresh BM, which had been collected in EDTA tubes, was processed within 6 hours after collection and stored at 4°C. Nucleated BM cells were separated over a discontinuous Percoll gradient. After Percoll gradient centrifugation, the cells were washed with phosphate buffered saline and resuspended in RPMI 1,640 medium with 0.1% bovine serum albumin. The cells were stained with antihuman CD41-allophycocyanin, and CD41 ^+^ MKs were separated using a FACS Aria cell sorter (BD Biosciences). After washing and centrifugation, 1 mL of Trizol (Life Technologies, Carlsbad, California, United States) was added and mixed thoroughly. After extracting total RNA, we used Nanodrop (Thermo Fisher Scientific, Waltham, Massachusetts, United States) and Qiaxcel (QIAGEN, Hilden, Germany) to detect the concentration and purity of the extracted RNA. The samples of total RNA (1 µg) were treated with Ribo-off ribosomal RNA (rRNA) Depletion Kit (Vazyme, Nanjing, China) before the RNA-sequencing libraries were constructed. The RNA-sequencing libraries were prepared using the VAHTS Total RNA-seq (H/M/R) Library Prep Kit for Illumina following the manufacturer's instructions (Vazyme, Nanjing, China). Annotations of mRNA in the human genome were retrieved from the GENCODE V29 ( https://www.Gencodegenes.org/human/release_19.html ). The genes that were differentially expressed between groups were analyzed using a *t* -test. The most differentially expressed genes were investigated for their involvement in Kyoto Encyclopedia of Genes and Genomes (KEGG) pathways ( https://www.genome.jp/kegg/ ) using the Database for Annotation, Visualization and Integrated Discovery (DAVID) v6.8 ( https://david.ncifcrf.gov/ ). The enriched pathways were filtered with *p* -values <0.01. Preranked gene set enrichment analysis (GSEA) was run on the ranked list using the Molecular Signatures Database (MSigDB) ( https://www.gsea msigdb.org/gsea/msigdb/) as the gene sets.

### Quantitative Reverse Transcription Polymerase Chain Reaction

Total RNA was extracted from CD41 ^+^ MKs using RNAiso Plus reagent (Takara, Shiga, Japan), and 1.5 µg total RNA from cultured cells was reverse transcribed using a PrimeScriptP RT Reagent Kit (Takara) according to the manufacturer's instructions. RT-qPCR was performed using a 7,500 Fast Real-Time PCR System (Applied Biosystems, Foster City, California, United States). The amplified transcript level of each specific gene was normalized to that of GAPDH.

#### Statistical Analysis

Statistical analysis was performed using SPSS statistics 24.0 (SPSS) and R statistical software. Results were expressed as means ± standard error. The Kolmogorov–Smirnov test was used to check for the normal distribution of data, and statistical differences between groups were observed using a *t* -test or the Mann–Whitney test. The Kaplan–Meier curve was performed and the log-rank test was applied to estimate and compare OS between the two groups. The level of significance was *p* < 0.05 for all analyses.

## Results

### Patient Cohort: Clinical Characteristics

**Fig. 1:**
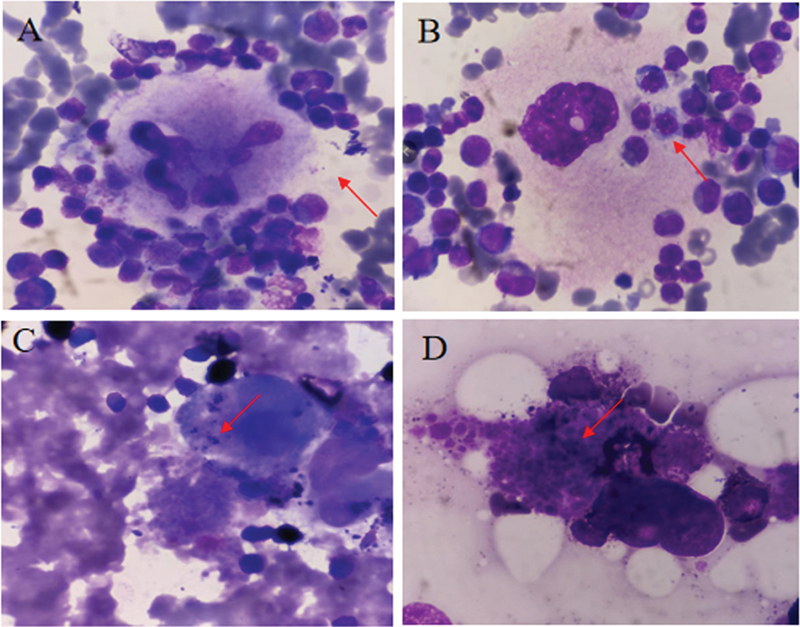
Emperipolesis in CD34 ^+^ CD61 ^+^ megakaryocytes (MKs). ( **A** ) The “rose cluster” phenomenon formed by MK and neutrophils ( *red arrow* ). ( **B** ) Neutrophils passing through the cytoplasm of MK ( *red arrow* ). ( **C** and **D** ) POX particles inside the cytoplasm of MK ( *red arrows* ).

The clinical characteristics of MDS patients were summarized in Table 1 . MDS subtypes according to the WHO 2022 classification included MDS with low blasts (39.42%), MDS with increased blasts type 1 (33.65%), MDS with increased blasts type 2 (12.5%), MDS hypoplastic (6.73%), MDS with fibrosis (5.77%), MDS with biallelic TP53 inactivation (0.96%), and MDS with low blasts and SF3B1 mutation (0.96%). The IPSS-R risk distribution was very high (15.38%), high (33.65%), intermediate (30.77%), and low (20.19%). Among 104 MDS cases, the median age at diagnosis was 64 years (range: 22–84 years). The median BM blasts, absolute neutrophil count, and hemoglobin were 5.00% (range: 0.19–17.00%), 1.78 × 10 ^9^ /L (range: 0.10–29.95 × 10 ^9^ /L), and 67.00 g/L (range: 9.00–153.00 g/L), respectively. Twenty-four cases (24/104, 23.1%) showed a positive expression of CD34 on MKs (28.00%, range: 21.00–80.00%), and 45.83% (11/24) of them had the emperipolesis phenomenon between MKs and neutrophils. In the BM of these patients, 20 to 50% of MKs contained neutrophils and neutrophil-specific myeloperoxidase-positive granules were found within the MK cytoplasm near emperipolesis neutrophils ( Fig. 1 ). During follow-up, 39 (39/104, 37.50%) deaths were recorded.

**Table 1:** Clinical characteristics of 104 MDS patients according to the MK mutation status

	With MK mutation ( *n* = 40) Median (Min–Max)	Without MK mutation ( *n* = 64) Median (Min–Max)	*p* -Value
Age, years	64.00 (22.00–79.00)	63.50 (22.00–84.00)	0.810
Male, *n* (%)	22 (55.00%)	43 (67.19%)	0.212
Neutrophil count, ×10 ^9^ /L	1.81 (0.10–15.05)	1.64 (0.13–29.95)	0.789
Hemoglobin, g/L	61.50 (37.00–100.00)	68.00 (9.00–153.00)	**0.023**
Platelet, ×10 ^9^ /L	77.50 (8.00–420.00)	102.00 (14.00–936.00)	**0.044**
BM blast, %	5.00(0.5–19.00)	5.00 (0.50–17.00)	0.826
CD34 ^+^ CD61 ^+^ MK, %	10.00 (1.00–72.00)	4.00 (1.00–80.00)	**0.012**
Emperipolesis, *n* (%)	8 (20.00%)	3 (4.69%)	**0.032**
Diagnosis, *n* (%)			0.452
MDS-h	4 (10.00%)	3 (4.69%)	
MDS-LB	16 (40.00%)	25 (39.06%)	
MDS-IB1	13 (32.50%)	22 (34.38%)	
MDS-IB2	5 (12.50%)	8 (12.50%)	
MDS-f	1 (2.50%)	5 (7.81%)	
MDS-SF3B1	0 (0%)	1 (1.56%)	
MDS-biTP53	1 (2.50%)	0 (0%)	
IPSS-R, *n* (%)			0.156
Very high	9 (22.50%)	7 (10.94%)	
High	16 (40.00%)	19 (29.69%)	
Intermediate	9 (22.50%)	23 (35.94%)	
Low	6 (15.00%)	15 (23.44%)	
OS, months	33.00 (3.00–65.00)	39.00 (12.00–78.00)	**0.013**

Abbreviations: BM, bone marrow; IPSS-R, International Prognostic Scoring Systems-Revised; Max, maximum; MDS, myelodysplastic syndromes (neoplasms); MDS-biTP53, MDS with biallelic TP53 inactivation; MDS-f, MDS with fibrosis; MDS-h, MDS hypoplastic; MDS-IB1/2, MDS with increased blasts type1/2; MDS-LB, low blasts; MDS-SF3B1, MDS with low blasts and SF3B1 mutation; Min, minimum; MK, megakaryocytes; OS, overall survival.

Bold: *p* <0.05.

### Genes Mutation Associated with Megakaryocytes in Myelodysplastic Syndrome

Gene mutation associated with MKs was found in 40 patients. The most frequent mutation included *TUBB1* (11.54%), *VWF* (8.65%), and *NBEAL2* (5.77%), and the common mutation was *RUNX1* (3.85%), *GFI1B* (3.85%), *JAK2* (3.85%), *ITGA2B* (2.88%), *ITGB3* (2.88%), *ANKRD26* (2.88%), *CD36* (1.92%), *GP9* (0.96%), *MPL* (0.96%), and *FLNA* (0.96%). *TUBB1* mutation was found in 12 cases, including eight single nucleotide variants: p.(R307H), *n* = 5; p.(Q43P), *n* = 3; p.(Q43H), *n* = 1; p.(L273F), *n* = 1; p.(I377L), *n* = 1; p.(G38R), *n* = 1; p.(T149P), *n* = 1; p.(R318W), and *n* = 1. In all MDS patients, *TUBB1* p.(R307H) and p.(Q43P) mutation was the most common point mutation. *NBEAL2* p.(S2054F), *VWF* p.(G1172V), *GFI1B* p.(L262P) mutation was found in six cases (5.77%), six cases (5.77%), and three (2.88%) cases, respectively.

When the clinical features of patients with or without MK mutation were compared, we found that patients with MK mutation showed similar myeloblast numbers (4.25 vs. 5.00%, *p* = 0.457) and absolute neutrophil count (1.81 ×10 ^9^ /L vs. 1.64 ×10 ^9^ /L, *p* = 0.789). However, they presented a higher proportion of CD34 ^+^ CD61 ^+^ MKs (10.00 vs. 4.00%, *p* = 0.012) and shorter OS (33.15 vs. 40.50 months, *p* = 0.013). We evaluated the relationship between MK mutation and CD34 ^+^ CD61 ^+^ MKs proportion and found that 16 patients with MK mutation showed a high proportion of CD34 ^+^ CD61 ^+^ MKs, and half of them had increased emperipolesis of neutrophils. Further, patients with MK mutation were divided into two subgroups (group A: CD34 ^+^ CD61 ^+^ MKs ≧20.00%, *n* = 16; group B: CD34 ^+^ CD61 ^+^ MKs <20.00%, *n* = 24). The average percent of CD34 ^+^ CD61 ^+^ MKs was 29.00% (range: 23.00–49.00%) and 4.00% (range: 2.00–6.00%). Patients in the group A had lower platelet counts (36.00 ×10 ^9^ /L vs. 88.50 ×10 ^9^ /L, *p* = 0.015), more profound emperipolesis (8 cases vs. 0 case, *p* = 0.001), and lower hemoglobin level (59.50 vs. 67.00 g/L, *p* = 0.030). Moreover, soluble S100A9 in BM fluids was elevated (group A vs. group B, 51.42 vs. 7.76 ng/mL, *p* < 0.001); notedly, patients with emperipolesis showed markedly higher S100A9 level (88.80 vs. 29.67 ng/mL, *p* = 0.010) than others without emperipolesis.

### Platelet Function

**Fig. 2:**
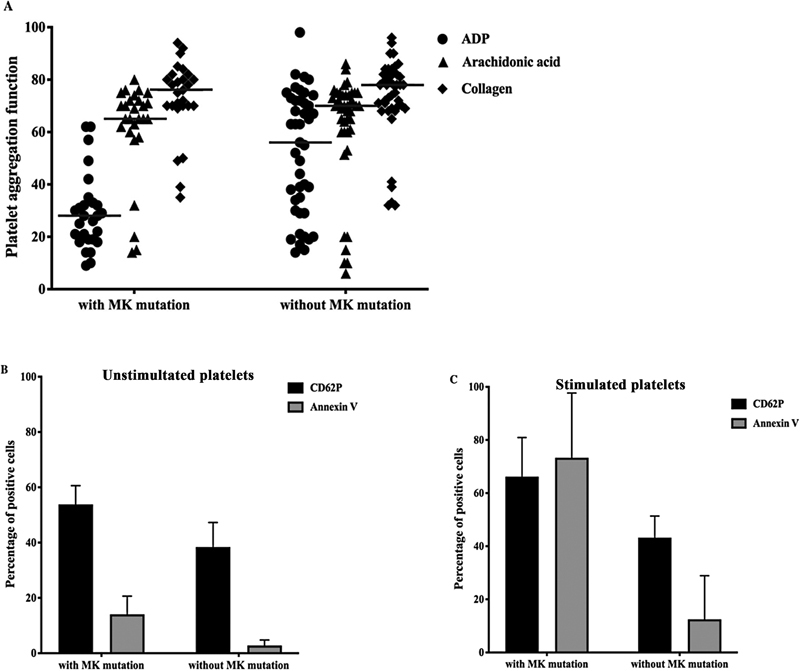
Platelet aggregation and activation ***.*** ( **A** ) Platelet aggregation function in myelodysplastic syndromes patients with or without megakaryocyte mutation. ( **B** ) The expression of CD62P and Annexin V on unstimulated platelet surface. ( **C** ) The expression of CD62P and Annexin V on stimulated platelet surface.

Platelet function was studied in 70 cases (platelet count: 105.50 ×10 ^9^ /L, range: 48.00–936.00 ×10 ^9^ /L). Platelet aggregation with ADP was defective in 38 patients (38/70, 54.29%), whereas aggregation with collagen or arachidonic acid was normal for all patients ( Fig. 2A ). In total, 22 patients with MK mutation (22/27, 81.48%) had ADP-induced platelet aggregation defects, and 13 of them (13/22, 59.09%) developed platelet transfusion refractoriness (PTR) during the first cycle of chemotherapy. Sixteen patients without MK mutation (16/43, 37.21%) had ADP-induced platelet aggregation defects, and two of them (2/16, 12.50%) developed PTR. To quantify the platelet reactivity, we measured the fraction of activated platelets before and after thrombin-induced stimulation. Unstimulated platelets from patients with MK mutation had a high expression of CD62P (53.50 vs. 41.10%, *p* = 0.001) and Annexin V (13.70 vs. 2.25%, *p* = 0.001) ( Fig. 2B ). When platelets from patients with MK mutation were activated by thrombin, they had a higher share of Annexin V on the surface (patients with MK mutation vs. patients without MK mutation, 85.80 vs. 6.40%, *p* = 0.002) ( Fig. 2C ).

### RNA-Sequencing of Megakaryocytes

**Fig. 3:**
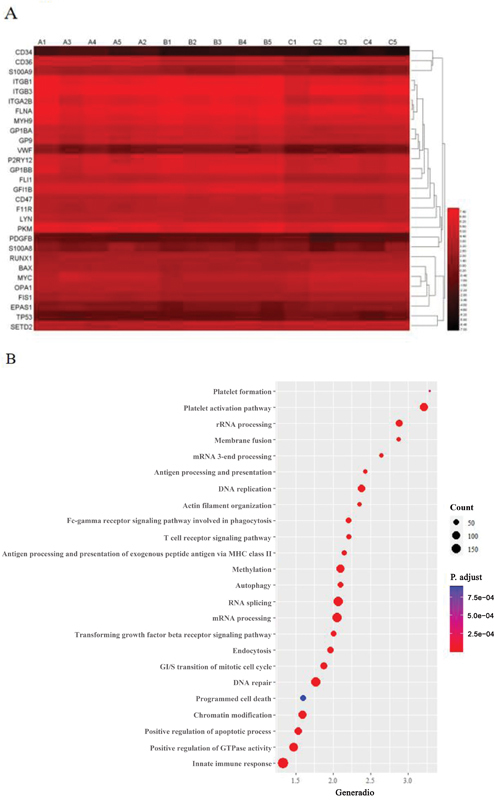
Characterization of protein-coding RNA in megakaryocytes (MKs). ( **A** ) A heatmap showing distinctly defined expression profiles of mRNA in MKs. ( **B** ) Kyoto Encyclopedia of Genes and Genomes pathway analysis in MKs.

**Fig. 4:**
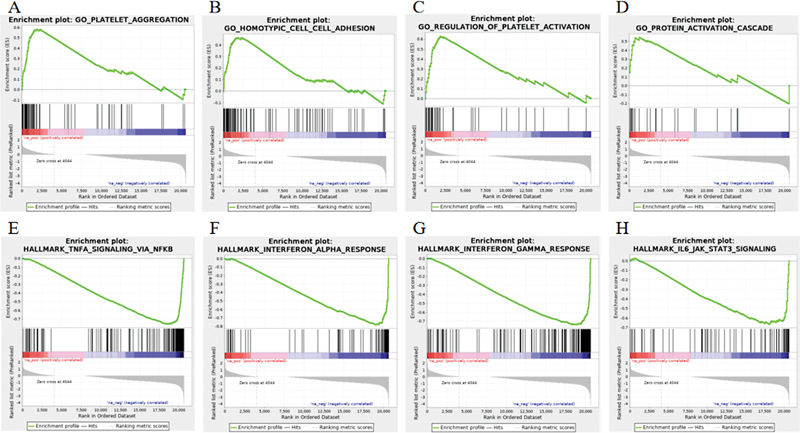
Enrichment plots from gene set enrichment analysis of MKs. ( **A** – **D** ) Gene set enrichment analysis (CSEA) results showing upregulated pathways. ( **A** ) Platelet aggregation, normalized enrichment score (NES) = 2.50, *p* < 0.0001. ( **B** ) Homotypic cell–cell adhesion, NES = 2.50, *p* < 0.0001. ( **C** ) Regulation of platelet activation. NES = 2.47, *p* < 0.0001. ( **D** ) Protein activation cascades NES = 2.09, *p* < 0.0001. ( **E** – **H** ) GSEA results showing downregulated pathways. ( **E** ) TNFA signaling, NES = −2.1, *p* < 0.0001. ( **F** ) Interferon alpha response, NES = −2.09, *p* < 0.0001. ( **G** ) Interferon-gamma signaling, NES = −2.03, *p* < 0.0001. ( **H** ) IL6-JAK-STAT3 signaling, NES = −1.79, *p* < 0.0001.

**Fig. 5:**
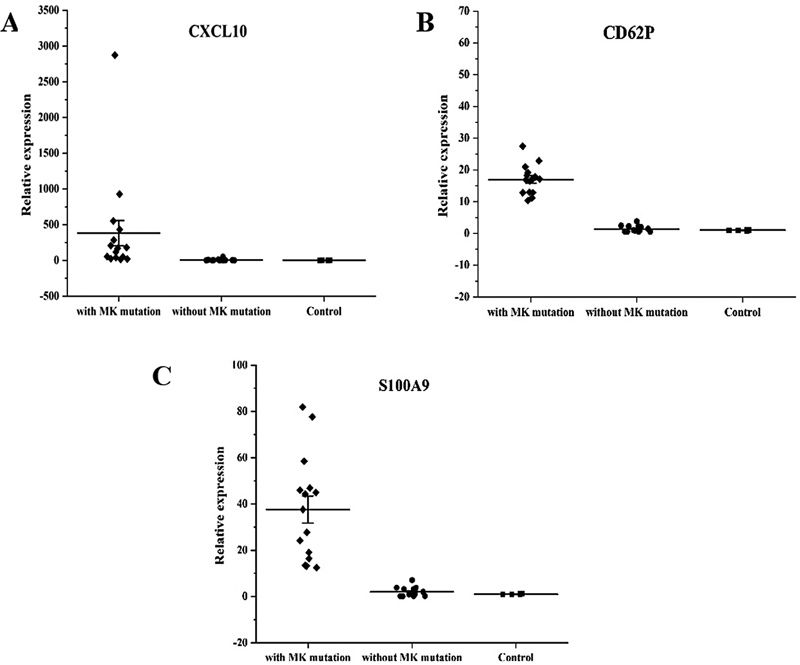
Reverse transcription polymerase chain reaction analysis of the potential gene targets in MDS with MK mutation. ( **A** ) The level of CXCL10 gene expression. ( **B** ) The level of CD62P gene expression. ( **C** ) The level of *S100A9* gene expression.

We further explored the transcriptional heterogeneity of specific MKs status. Among 10,643 mRNA that were detected, 4,826 were differentially expressed in patients with MK mutation. Within these 4,826 mRNA, 269 were continuously upregulated and 4,557 continuously downregulated in group A. Gene ontology (GO) analyses suggested that the differentially expressed genes were associated with chromatin modification, DNA repair, transcriptional regulation, programmed cell death, and other important functions. Further investigation of these processes showed that platelet formation and function were the core processes of the GO tree ( Fig. 3A ). Increased expression of MKs genes, not just in the intracellular proteins (PKM, VWF, and FLNA), but also cell surface antigens (CD36, ITGB1, and ITGB3), was noted. KEGG pathway analyses suggested that innate immune responses, RNA splicing, and mRNA processing were most enriched among the differentially expressed genes ( Fig. 3B ). GSEA analysis identified that platelet function, platelet aggregation, and platelet activation pathways were upregulated the most ( Fig. 4 ). We focused on several candidate mRNA and verified the changes in their expression levels, and qRT-PCR assays showed that levels of CXCL10, CD62P, and S100A9 were remarkably increased ( Fig. 5 ).

## Discussion

MDS is characterized by multiple clonal hematopoietic defects, and some patients may present with isolated thrombocytopenia and megakaryocytic dysmorphia or atypia. The development and differentiation from MKs to platelets are revealed to be a complex process that can be driven by a number of genes. With the advent of next-generation sequencing, an increasing number of genes associated with megakaryocytopoiesis have been elucidated. In our present study, the most frequent mutation in MDS included *TUBB1* , *NBEAL2* , and *VWF* gene. *TUBB1* mutation was commonly found to disrupt the normal assembly of microtubules and contributed to the accumulation of DNA damage and genetic instability. 16 17 *NBEAL2* mutation was associated with a genetic disturbance of MK differentiation, with 36 to 65% of MKs containing neutrophils. 18 19 *VWF* mutation resulted in a reduced number of platelets by MKs, the ectopic release of platelets in the BM, and the increased clearance of platelet-VWF complexes. 20 Regardless of age and IPSS-R score, patients with MK mutation were at increased risk of developing thrombocytopenia and/or platelet dysfunction during their lifetime. 21 22 Therefore, early recognition of MK mutation in MDS patients could permit appropriate treatment and adequate monitoring for disease progression.

MK mutation was associated with high levels of CD34 on MK, which was likely a result of dysplastic maturation committed to the megakaryocytic lineage. Indeed, previous studies had documented that *GATA1* , *TP53* , and *RUNX1* genes were related to the high-level expression of CD34 on MKs. The most recently discovered gene was *GFIB* , which repressed the CD34 promoter at a direct transcriptional level, and this repression was attenuated by gene mutation. 23 24 25 The enhanced emperipolesis in patients with a high percent of CD34 ^+^ CD61 ^+^ MKs was of interest. Petzold et al confirmed that neutrophils can “pluck” on MKs to tune platelet release in BM. In Pierre Cunin's model system, they demonstrated that neutrophil membranes transfer to MKs' demarcation membrane system during emperipolesis. Receptor–ligand pairs mediating the observation between neutrophils and MKs have been found in many studies, such as CXCR4–CXCL12, CD18–ICAM-1, or PSGL-1–CD62P. 26 27 28 29 Our data found that CD34 ^+^ CD61 ^+^ MKs with emperipolesis exhibited hyperreactivity and immature immunity, epitomized by increased CD62P, CXCL10, and S100A9 transcripts. Together these results implied a mechanistic role of emperipolesis on MK differentiation, and defective platelet formation maybe associated with “pathological emperipolesis” in MDS patients with high levels of CD34 ^+^ CD61 ^+^ MKs.

Bleeding complications, as a major cause of morbidity and mortality, are commonly seen in MDS patients. There may be bleeding episodes of varying severity, and there are variable platelet aggregation defects correlated with poor prognosis. A study of 75 MDS cases showed defective platelet activation and increased apoptotic platelets consistent with defective platelet production. 10 Another study observed that ADP was one of the most common agonists with the platelet aggregation defect and confirmed that defective platelet aggregation was strongly related to MDS of worse prognosis. 11 These results were consistent with our findings in patients with MK mutation, especially with high levels of CD34 ^+^ CD61 ^+^ MKs. These patients showed a decrease in ADP-induced platelet aggregation and an increase in platelet proactivation and apoptotic platelets. Managing bleeding due to dysfunctional platelets was based on general principles, and the most studied intervention was the transfusion of “normal” platelets. 30 However, it may not be effective in major bleeding, for example, intracranial hemorrhage. To make matters worse, one study suggested that platelet transfusion was instead associated with higher rates of adverse events and death. 31 In the present study, PTR was increased for MDS patients with MK mutation. Future studies will expand the sample size and focus more on exploring the relationship between MK mutation and the molecular etiology of PTR.

In summary, our study was performed to characterize a poor prognostic factor in MDS patients. We presented evidence that (1) the most common point mutation was *TUBB1* p.(R307H) and p.(Q43P), followed by *NBEAL2* p.(S2054F) and *VWF* p.(G1172V); (2) MK mutation was associated with the high percent of CD34 ^+^ CD61 ^+^ MKs; (3) platelet formation and platelet function were commonly affected by MK mutation; (4) CD62P, CXCL10, and S100A9 may be the potential targets in patients with MK mutation. Further studies are no doubt required to evaluate the molecular link between gene defects and platelet production and to establish any prognostic value.
